# An *ab initio* study of the polytypism in InP

**DOI:** 10.1038/srep33914

**Published:** 2016-09-26

**Authors:** Luis C. O. Dacal, A. Cantarero

**Affiliations:** 1 Instituto de Estudos Avançados, IEAv-CTA, PO Box 6044, 12228-970, São José dos Campos - SP, Brazil; 2 Molecular Science Institute, University of Valencia, PO Box 22085, E46071 Valencia, Spain

## Abstract

The existence of polytypism in semiconductor nanostructures gives rise to the appearance of stacking faults which many times can be treated as quantum wells. In some cases, despite of a careful growth, the polytypism can be hardly avoided. In this work, we perform an *ab initio* study of zincblende stacking faults in a wurtzite InP system, using the supercell approach and taking the limit of low density of narrow stacking faults regions. Our results confirm the type II band alignment between the phases, producing a reliable qualitative description of the band gap evolution along the growth axis. These results show an spacial asymmetry in the zincblende quantum wells, that is expected due to the fact that the wurtzite stacking sequence (ABAB) is part of the zincblende one (ABCABC), but with an unexpected asymmetry between the valence and the conduction bands. We also present results for the complex dielectric function, clearly showing the influence of the stacking on the homostructure values and surprisingly proving that the correspondent bulk results can be used to reproduce the polytypism even in the limit we considered.

Indium phosphide is a III-V semiconductor that crystallizes in the zincblende (ZB) phase in bulk, but that shows the wurtzite (WZ) crystal structure for nanowires^1^. Actually, the symmetry of these low dimensional structures can be controlled in great extent through the growth conditions^2^,^3^. Some works show a relation between phase purity and the nanowires radius^4^ while others state that the stacking sequence of newly grown monolayers are influenced by the underlying monolayers^5^. As a result, the fine tuning of the growth conditions can give rise to superlattices of the two polytypes or twin planes along the nanowire axis^6^,^7^. Another interesting subject is the study of the influence of the stacking faults on the nanowire properties what opens the possibility of engineering them through a strict structural control^8^,^9^.

On the theoretical side, some attempts have been made to estimate the band offset between the WZ and ZB regions in polytypic homostructures mainly through the connection between bulk calculations^10^,^11^,^12^. Recently, the ***k*** · ***p*** method was extended to these homostructures^13^,^14^,^15^ demanding large unit cells for a good description of the systems.

A better understanding of the InP properties in real nanowire samples is essential to increase their potential as building blocks of nanoscale electronic and photonic devices^16^,^17^. In order to improve this understanding, we present here *ab initio* calculations of the electronic properties of WZ InP samples with stacking faults with the ZB symmetry. To the best of our knowledge, it is the first time that a sample with stacking faults is modeled using the supercell approach. This gives rise to a more realistic description of the system than using bulk results^10^,^11^,^12^. For example, with this approach, we can show how the band gap evolves along the growth axis, avoiding the artificial abrupt band offset commonly assumed at the WZ-ZB interface^10^,^11^,^12^,^13^,^14^,^15^. Remembering that the ZB stacking faults can be hardly avoided when one wants to grow a pure WZ InP nanowire, in our model, the ZB regions are far from each other, simulating a WZ InP sample with low density of narrow ZB layers.

## Method

**Figure 1:**
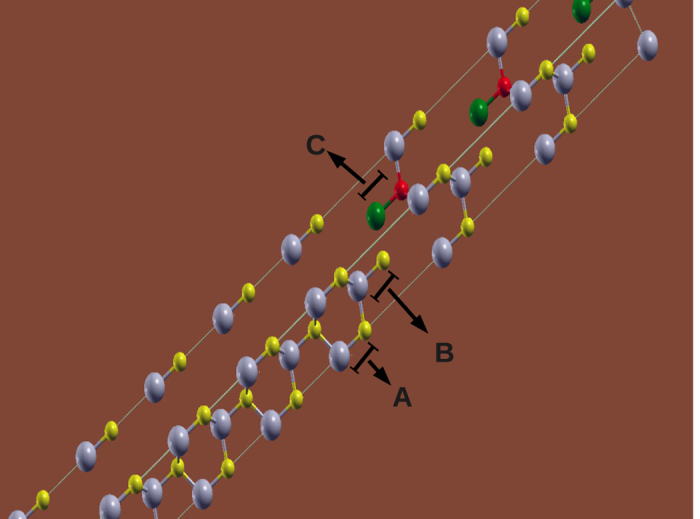
Schematic representation of the interface between WZ and ZB regions in our supercell. The A, B and C layers whose stacking sequence defines the region symmetry are indicated. The atoms in A and B layers are in gray and yellow. The C layer that is present only in the ZB region presents atoms in green and red.

In order to model an InP sample in the WZ phase with a low density of ZB stacking faults, we built up an hexagonal supercell with 15 layers of In and P atoms in the AB stacking sequence along the hexagonal *c* axis of the WZ (4 atoms per layer, 60 atoms in total). On the top of this supercell, we added three layers of In and P atoms using the ABC stacking sequence as along the [111]–direction of their ZB phase (6 atoms per layer, 18 atoms in total). In other words, we built up an hexagonal supercell superimposing 15 InP WZ cells along their *c* axis and over them we put 3 InP ZB cells where the cubic [111]–direction corresponds to the hexagonal *c* axis. In Fig. 1 we show an schematic representation of the interface between WZ and ZB regions in our supercell. The A, B and C layers whose stacking sequence defines the region symmetry are indicated. The C layer that is present only in the ZB region has a different color scheme for clarity.

We define a low density of ZB stacking faults as a case where the density of states (DOS) for the atoms at the center of the WZ region reproduce the results obtained for the bulk WZ system. This condition intend to guarantee that the WZ bulk is reproduced at the middle between two neighboring ZB regions. Our results show that this condition is achieved for our WZ region formed by 15 cells. On the other hand, since at the WZ-ZB interface the ABC sequence contains an AB region, we need to use at least 3 ZB cells to assure the existence of a well defined ZB region.

The *ab initio* calculations have been performed using the “*Linearized Augmented Plane Wave method*” (LAPW) as implemented in the Wien2k code^18^. Our basis functions were expanded up to *R*_*mt*_ × *K*_*max*_ = 6, where *R*_*mt*_ is the smallest of the atomic “muffin-tin” radii and *K*_*max*_ is the magnitude of the largest *K* vector for the plane wave basis functions. The atomic “muffin-tin” radii used here were 2.50 Bohr for In and 2.11 Bohr for the P atoms. We have employed the modified Becke-Johnson exchange potential plus LDA correlation with its original parametrization (P-original)^19^,^20^. The energy separation for core and valence states was −6.0 Ry and the spin-orbit coupling was taken into account during the calculation of the electronic properties. The only difference among the supercell and bulks calculations was the K-grid. For the total energy and DOS calculations we employed K-grids with 12, 16 and 10 inequivalent points in the irreducible part of the Brillouin Zone (BZ) for the supercell, WZ and ZB bulks respectively. To obtain the dielectric functions, denser grids were used (see below).

The structural parameters used for the InP cells in the WZ and ZB phases were previously optimized for the bulk cases^21^. The supercell were built up as described above and we considered two cases. In the first case, on top of the 15 optimized WZ cells, we added three ZB cells with the same WZ *a* parameter. The volume per atom obtained for the optimized ZB bulk was recovered through changes in the atomic distances along the *c* axis. In the second case, we kept the same atomic distances along the *a* and *c* axes found in the optimized WZ bulk and changed only the stacking sequence from AB to ABC.

In both cases we got little forces on atoms along the *c* axis, proving that we were dealing with relaxed supercells. More specifically, these forces were always lower than 2.9 mRy/Bohr in the second structure and we have chosen this structure for our study. Besides that, these results indicate that strain effects must have a minor role on the electronic properties of the supercell.

## Results

**Figure 2:**
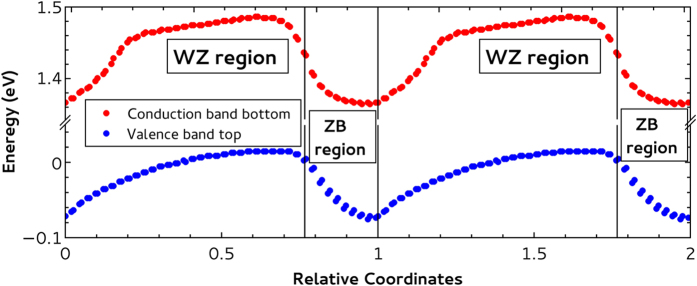
Top of the valence band (blue circles) and bottom of the conduction band (red circles) as a function of the atomic relative coordinates along the *c* axis for two consecutive WZ InP supercells with ZB stacking faults. The WZ and ZB regions are indicated in the graph.

In Fig. 2 we show how the top of the valence band (blue circles) and the bottom of the conduction band (red circles) evolve as a function of the atomic relative coordinate along the *c* axis for two consecutive WZ InP supercells with ZB stacking faults. To obtain these values, we compare the DOS per atom in the supercell with that of the bulk systems. We have observed that at the center of the WZ region, the corresponding bulk DOS was reproduced at the band gap neighborhood. After that, we took from these central atoms the first derivative of the DOS at zero energy (top of the valence band) and at the gap energy of the WZ bulk (bottom of the conduction band) and used them to determine how the two band extrema evolve along the supercell *c* axis. Obviously, this is not a rigorous approach and Fig. 2 should be taken as a qualitative description. Despite of this, our results show a smooth transition between the WZ and ZB regions, what is a more realistic description than the commonly assumed abrupt transition.

As one can see, our results confirm the type II band alignment with holes concentrated at the WZ side of the WZ → ZB interface, while the electrons tend to be at the ZB side of the ZB → WZ interface. This carriers spatial separation has a significant impact on the exciton recombination time^8^ and can be tuned through a strict control of the atomic stacking sequence during the sample growth. We can also notice that the WZ → ZB and ZB → WZ are not equivalent due to the lack of mirror symmetry in the supercell and at the ZB region. Finally, the shape of the band extrema evolution along the supercell *c* axis is different for the valence and conductance bands. This is in a clear disagreement with the commonly modeled type-II quantum wells, since, in our results, the conduction band profile looks like a square quantum well, while the valence band one is similar to a teeth saw quantum barrier.

**Figure 3:**
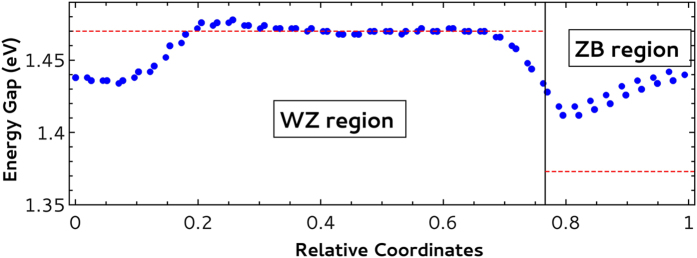
Energy gap as a function of the atomic relative coordinates along the *c* axis of the WZ InP supercell with ZB stacking faults. The WZ and ZB regions are indicated. The dashed lines show the energy gap of the correspondent bulk system.

In Fig. 3, we show the energy band gap (the difference between the two curves in Fig. 2) as a function of the atomic relative coordinates along the *c* axis of the supercell. Here, it is important to say that we have reduced the basis size when compared with our previous work^21^. The main change was the reduction of *R*_*mt*_ * *K*_*max*_ from 9 to 6 in order to decrease the computation time, but keeping an acceptable level for results quality. In order to get a fair comparison with the bulk systems, we repeated the correspondent calculations with the same parameters used for the supercell and we observed a reduction of approximately 23 meV in the bulk gaps when compared with the previously published values^21^. Here we obtain 1.470 eV and 1.373 eV for the WZ and ZB gaps, respectively (dashed lines in Fig. 3).

As previously commented, the calculated DOS at the center of the WZ region reproduced the WZ bulk results and we took their derivative as a marker for the top of the valence band and the bottom of the conduction band. As a result, Fig. 3 shows the exact reproduction of the WZ bulk gap at the center of WZ supercell region.

It is also important to notice that the band gap, as the difference between the two curves in Fig. 2, reinforces the lack of mirror symmetry in the supercell with an almost linear behavior inside the ZB region and very distinct profiles at the ZB → WZ and WZ → ZB interfaces. The impact of this asymmetry in the carriers localization and lifetime has been experimentally investigated^8^.

**Figure 4:**
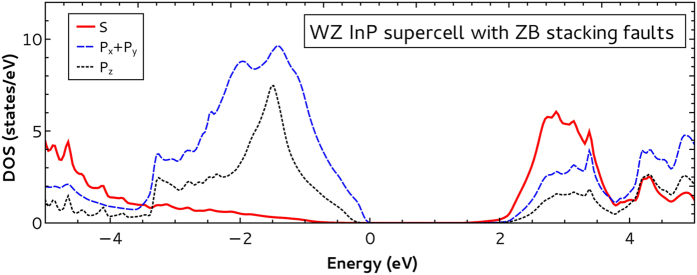
Projected density of states as a function of energy for the WZ InP supercell with ZB stacking faults. We present results for *S* (full red line), *P*_*x*_ + *P*_*y*_ (dashed blue line) and *P*_*z*_ (dotted black line) projections.

Figure 4 shows our projected density of states results for the WZ InP supercell with ZB stacking faults. We present the calculated values for *S* (full red line), *P*_*x*_ + *P*_*y*_ (dashed blue line) and *P*_*z*_ (dotted black line) projections. As one can see, the WZ symmetry results are reproduced^21^. This means that the ZB region has practically no influence in the polarization properties of the system as experimentally verified for the limit of low density of ZB stacking faults^22^.

The convergence of the dielectric function was obtained with a more dense K-grid. More specifically, we used 90, 198 and 104 inequivalent points in the irreducible part of the Brillouin Zone for the supercell, WZ and ZB bulks, respectively.

**Figure 5:**
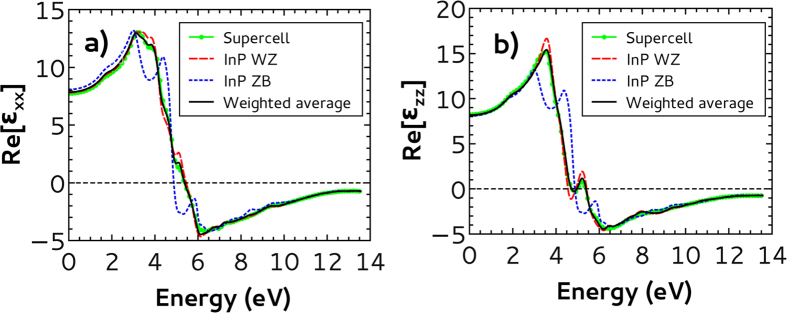
Real part of the complex dielectric function as a function of incident photon energy for polarizations in the *xy* plane (a) and along the *z* axis (b). We show the results for the WZ InP supercell with ZB stacking faults (full green line with dots), WZ bulk (dashed red line) and ZB bulk (dotted blue line). We also show the weighted average between WZ and ZB bulk results (5:1) (full black line).

In Fig. 5, we present the real part of the complex dielectric permittivity as a function of the incident photon energy for the WZ InP supercell with ZB stacking faults (full green line with dots), WZ bulk (dashed red line) and ZB bulk (dotted blue line). In the left panel (a), we consider light polarization in the *xy* plane and, in the right panel (b), the polarization is taken along the *z* axis. As one can see, the ZB stacking faults have a small effect on the real part of the supercell dielectric function. The most noticeable deviations from the WZ results occur around 4 and 5 eV for both polarizations where the ZB values present pronounced dips. Nevertheless, it is important to show that a weighted average between the WZ and ZB bulk results perfectly fits the supercell ones, even for the points where the supercell results exhibit the above mentioned deviation from the WZ values. The full line in both panels represent this average with weight 5 for WZ and 1 for ZB results (the rate between the number of bulk cells in the supercell). This is somehow surprising because we did not guarantee that the ZB region was able to reproduce the correspondent bulk environment. It indicates that strain effects, that should differentiate the stacking faults from the bulk, must have a minor contribution to the supercell properties. Beside this, it is expected that the interfaces between the symmetries have a significant role considering the narrow ZB region as reflected in the results for the conduction and valence band edges evolving along the supercell *c* axis (Figs 2 and 3). In conclusion, we can say that this somehow counterintuitive result indicates that approximations that consider abrupt transitions between the phases can be suitable even in the case of low density of narrow stacking faults regions, opening the possibility of avoiding computationally expensive supercells when modeling dielectric functions in homostructures.

**Figure 6:**
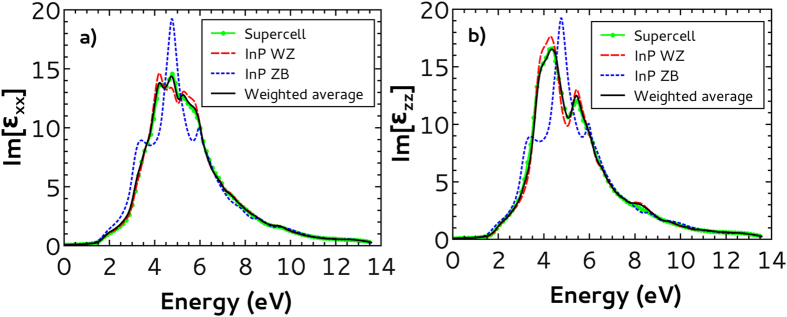
Imaginary part of the complex dielectric function as a function of incident photon energy for polarizations in the *xy* plane (a) and along the *z* axis (b). We show the results for the WZ InP supercell with ZB stacking faults (full green line with dots), WZ bulk (dashed red line) and ZB bulk (dotted blue line). We also show the weighted average between WZ and ZB bulk results (5:1) (full black line).

Figure 6 is equivalent to Fig. 5 but for the imaginary part of the dielectric function. Here, the main contribution of the ZB stacking faults to the supercell results occurs in the energy interval from 4 to 6 eV for both polarizations, where the WZ and ZB bulk results are significantly different. Once more, the weighted average results perfectly fit the supercell ones even in the energy range where the ZB stacking faults has the most important contribution. This corroborates our previous conclusion that, surprisingly, bulk results can be used to reproduce supercell ones even for low densities of narrow stacking faults regions.

## Conclusions

Our *ab initio* analysis of a WZ InP system with ZB stacking faults in the limit of low density of narrow ZB regions confirmed the type II band alignment between the phases, what can lead to the control of the exciton lifetime in this kind of sample. Our results also show a reliable picture of the band gap smooth transition between the phases, with the ZB region presenting a linear dependence of the band gap with the position along the supercell *z* axis. Despite of this, the complex dielectric function results indicate the possibility of using bulk results to model the supercell systems. In other words, models that consider abrupt transitions between the phases retain the essential characteristics of this kind of systems.

## Additional Information

**How to cite this article**: Dacal, L. C. O. and Cantarero, A. An *ab initio* study of the polytypism in InP. *Sci. Rep.* **6**, 33914; doi: 10.1038/srep33914 (2016).
